# Patterns in exercise behaviour across pregnancy: a retrospective cohort study of physically active individuals from pre-conception to postpartum

**DOI:** 10.1007/s00421-026-06160-6

**Published:** 2026-02-25

**Authors:** Kate L. Oxnard, Rich D. Johnston, Jemima G. Spathis, Evelyn B. Parr, Kassia S. Beetham

**Affiliations:** 1 School of Behavioural and Health Sciences, Australian Catholic University, 1100 Nudgee Road, Banyo, Brisbane, Banyo, QLD 4014 Australia; 2 Sports Performance, Recovery, Injury and New Technologies (SPRINT) Research Centre, Australian Catholic University, 1100 Nudgee Road, Banyo, Brisbane, QLD 4014 Australia; 3 College of Healthcare Sciences, James Cook University, 1 James Cook Drive, Douglas, Townsville, QLD 4814 Australia; 4 Carnegie Applied Rugby Research (CARR) Centre, Carnegie School of Sport, Leeds Beckett University, Leeds, UK; 5 Mary MacKillop Institute for Health Research, Australian Catholic University, Level 3, 250 Victoria Parade, Fitzroy, VIC 3065 Australia

**Keywords:** Exercise behaviour, Active women, Training load, Wearable data, Antenatal

## Abstract

**Purpose:**

The safe upper limits of weekly exercise participation during pregnancy, as well as the modifications active individuals make to their weekly exercise behaviours while pregnant, are unclear. This retrospective observational study aimed to determine how active individuals modify their training behaviours before, during, and after pregnancy.

**Methods:**

We analysed data from 21 participants (aged 26–41 at the time of survey completion) who were physically active prior to their most recent pregnancy. Participants shared the exercise data recorded using a fitness application or monitoring device before, during, and after pregnancy. Participants also completed surveys about maternal and neonatal health outcomes. We used mixed-effects models to analyse weekly changes in participants’ total exercise duration, session duration, exercise frequency, and aerobic exercise intensity and volume.

**Results:**

Most participants (*n* = 19) exercised above recommended weekly durations (i.e., > 300 min.wk^− 1^) during at least one week of pregnancy. However, weekly exercise duration progressively decreased throughout the antenatal period, and increased during the first 12 weeks postpartum. All infants were born within the normal birthweight range (2500 to 4500 g), and 14 were born at full term (early term *n* = 5, late preterm *n* = 2).

**Conclusion:**

Our findings highlight that active pregnant individuals exceed weekly exercise recommendations, yet adapt their training load throughout pregnancy. These findings emphasise the importance of considering physical activity recommendations as relative in nature, and interpreting them in the context of previous exercise behaviours. Exercise professionals should provide individualised support to active individuals in their pregnancy and postpartum journeys.

**Supplementary Information:**

The online version contains supplementary material available at 10.1007/s00421-026-06160-6.

## Introduction

Pregnancy can be challenging for active individuals who wish to continue their training routines (Santos-Rocha and Szumilewicz 2024). While the safety and benefits of moderate- to vigorous-intensity exercise are well-established (Beetham et al. 2019; Melzer et al. 2012), people who engage in higher volumes and intensities of exercise face uncertainty about how to safely continue their sporting activities while pregnant (Davenport et al. 2022; Santos-Rocha and Szumilewicz 2024). This uncertainty may be further confounded by the inconsistent recommendations provided in global public health guidelines for exercise during pregnancy (Hayman et al. 2023; Worska et al. 2024). The 2021 Australian guidelines are the only recommendations that include a specific range of weekly exercise durations during pregnancy: 150 to 300 min of moderate-intensity exercise, 75 to 150 min of vigorous-intensity exercise, or an equivalent combination of both (Brown et al. 2022; Wieloch et al. 2022). Individuals who wish to exceed these weekly recommendations, or complete individual exercise sessions lasting for longer than 60 min, are encouraged to seek individual recommendations from their healthcare providers (Australian Institute of Sport 2025; Brown et al. 2022; Wieloch et al. 2022). Significant gaps in current knowledge concerning the safe upper limits of antenatal exercise participation limit the provision of effective exercise counselling for highly active pregnant individuals (Davenport et al. 2022; Okafor and Goon 2021; Worska et al. 2024).

There is ongoing concern about the possibility that engaging in excessive antenatal exercise may increase the risk of adverse pregnancy outcomes such as small for gestational age (Bisson et al. 2016). In 2017, an International Olympic Committee (IOC) expert group identified a need for research investigating how training changes during pregnancy for athletes in different sports, and how continued training may influence fetal growth and development (Bø et al. 2016). Since 2017, observational studies investigating antenatal exercise behaviours have typically utilised customised self-report questionnaires to collect training data, since no standardised exercise questionnaires are currently available for use with pregnant athletes (Beilock et al. 2001; Bø and Backe-Hansen 2007; Franklin et al. 2017; Kuhrt et al. 2018; Sigurdardottir et al. 2019; Sundgot-Borgen et al. 2019; Tenforde et al. 2015). The quality of participants’ responses to these customised questionnaires has interfered with attempts to fully describe antenatal exercise characteristics (Sundgot-Borgen et al. 2019). While a 2025 study found that engaging in long-duration antenatal exercise (> 300 min of moderate-to-vigorous intensity exercise per week) was associated with reduced odds of delivery complications and increased odds of experiencing diastasis recti abdominis in the postpartum period, the statistical analysis was restricted to exercise completed in the third trimester of pregnancy (Bains et al. 2025). As such, the safety of exceeding current weekly exercise recommendations (e.g., exercising for more than 300 min at moderate-intensity, or 150 min at vigorous-intensity, per week) throughout gestation remains unclear.

Fitness applications (‘apps’), such as Strava, and wearable devices, like Fitbit and Garmin, offer a promising alternative to questionnaires for investigating antenatal exercise behaviours and reliably capturing training data (Huhn et al. 2022). These technologies enable users to record standardised exercise data which includes, but is not limited to, the frequency (in sessions per week), intensity (using rating of perceived exertion and/or heart rate data), duration (in minutes per session), and modality (such as running and cycling) of exercise completed (Strava 2021). Because fitness apps and wearable devices have high rates of utilisation (Peake et al. 2018) and store activity data in standardised formats, they may provide a valuable source of exercise data for use in research (Bains et al. 2024; Best and Braun 2017). For example, a 2024 case study used Garmin Connect data to retrospectively analyse the weekly exercise behaviours of an ultramarathon runner across two consecutive pregnancies to explore trends in the participant’s weekly running duration, intensity, and distance across pre-conception, pregnancy, and the postpartum period (Bains et al. 2024). While the possibility of conducting research using fitness app data has been demonstrated, to our knowledge, no studies have used retrospective app data to investigate antenatal exercise behaviours in larger samples of active individuals. Leveraging fitness app data from larger numbers of participants may provide new insights into antenatal exercise behaviours, while reducing the risk of survey-related concerns such as recall bias.

The primary aim of this study was to describe how active individuals modify their exercise behaviours from 12 weeks prior to conception through to 12 weeks postpartum. We aimed to report how participants modified the weekly duration, frequency, intensity, and modality of exercise completed throughout the pre-conception to postpartum study period. We expected to observe progressive reductions in exercise intensity and duration throughout pregnancy, with recovery in the postpartum period.

## Methods

The methods are presented in alignment with checklist items outlined in the Strengthening the Reporting of Observational Studies in Epidemiology (STROBE) Statement for cohort studies (Vandenbroucke et al. 2007; von Elm et al. 2007). All participants provided written informed consent prior to commencing the study. The study protocol was approved by the Australian Catholic University Human Research Ethics Committee (HREC ID: 2022-2854E).

### Study design and eligibility

A retrospective cohort study design was implemented. Individuals who were at least 18 years of age and self-reported using a fitness app or wearable device to record details of at least 75% of exercise sessions completed during their most recent pregnancy were eligible to participate. This threshold was determined based on the methods used in previous studies investigating the safety of antenatal exercise interventions, where ≥75% session completion was considered indicative of ‘high participation’ (Backhausen et al. 2017), and used as an inclusion criterion for per-protocol statistical analysis (Acosta-Manzano et al. 2019). In addition to having recorded ≥75% of their completed antenatal exercise sessions, participants must have also self-reported being recreationally active (defined as completing at least 150 min of moderate-intensity exercise in most weeks (Brown et al. 2022; McKay et al. 2022)), or having competed in sport at any level in the six months prior to the conception of their most recent pregnancy.

### Recruitment and follow-up

Participants were recruited through social media advertisements and local and professional networks between May 2023 and September 2024. Participants accessed the online participant information letter, eligibility survey, and informed consent form using a website address or Quick Response code provided in the study advertisements. After providing written informed consent, participants who had not completed all study surveys received reminder emails once per week for a total of four weeks. Participants who completed all study surveys but did not upload any exercise data received two follow-up emails and were excluded from the analysis if no response was received.

### Outcomes

The study outcomes were participants’ weekly exercise duration (including all completed exercise modalities, in minutes per week), session exercise duration (in minutes per session), weekly exercise frequency (in sessions per week), aerobic exercise intensity (estimated by expressing participants’ average heart rate [HR] during each exercise session as a percentage of their age-predicted maximum HR), aerobic exercise volume (in metabolic equivalent minutes per week [MET-min·wk^− 1^]), and exercise modality (e.g., aerobic or resistance training). We did not use HR data to estimate resistance training intensity or volume, as HR may not provide an accurate representation of energy cost during resistance exercise above low intensity (≥ 30% one repetition maximum) (Reis et al. 2019). As such, resistance training intensity and volume were not analysed in the present study.

Participants’ maximum HR (HR_max_) values were predicted using the Fox formula (220 - age) (Fox et al. 1971). For participants who recorded their HR during exercise, weekly aerobic exercise volume was calculated by assigning a MET value to each exercise session based on the session intensity (average %HR_max_) (Brown and Bauman 2000). The assigned MET values for each exercise intensity aligned with those reported in the 2010 Exercise and Sports Science Australia (ESSA) and Fitness Australia position statement on exercise intensity terminology (Norton et al. 2009). The estimated energy expenditure (in METs) for each exercise session was then multiplied by the session duration (in minutes) to determine the exercise volume accumulated during the session (Garber et al. 2011). The volumes of all aerobic exercise sessions completed during a gestational week were combined to give each participant’s estimated weekly aerobic exercise volume (Garber et al. 2011). To facilitate the comparison of participants’ weekly exercise participation to relevant guidelines, the recommendations included in the 2021 Australian Guidelines for Physical Activity in Pregnancy (i.e., engaging in 150–300 min of moderate-intensity exercise, 75–150 min of vigorous-intensity exercise, or an equivalent combination of both, per week) were converted to an exercise volume of 450–900 MET-min·wk^− 1^ (Brown et al. 2022).

Participants’ self-reported exercise session modalities were reviewed in duplicate by two authors (KLO and KSB), and categorised as ‘aerobic’, ‘resistance’, ‘yoga’, or ‘other’. Any conflicts were resolved through discussion between the two authors. The American College of Sports Medicine (ACSM) define aerobic exercise as ‘any activity that uses large muscle groups, can be maintained continuously, and is rhythmic in nature’ (Patel et al. 2017), and resistance exercise as any ‘physical activity that employs exercise of a muscle, or group of muscles, against external resistance with the final goal of improving muscular strength, endurance, or power’ (Intzandt et al. 2018). Exercise sessions that were reported as ‘yoga’ were classified as such and were not included in the aerobic exercise intensity or volume analyses. Activities recorded as ‘warm-up’, ‘cool-down’, and ‘manual labour’ were removed prior to the analysis to ensure a focus on planned, structured, and repetitive exercise rather than preparatory or incidental activities.

Additional data collected included birthweight (defined as the first weight recorded after birth (Cutland et al. 2017)), gestational age at birth (defined as the number of completed weeks of gestation at the time of delivery (*Mosby’s dictionary of medicine*,* nursing & health professions*, 2014),) gestational weight gain (GWG; defined as the difference between body weight measured pre-conception and the last available body weight measured during pregnancy (Rasmussen and Yaktine 2009)), participants’ self-reported incidences of pregnancy-related symptoms and health conditions (e.g., urinary incontinence, gestational diabetes mellitus) and birth complications (e.g., perineal tearing). We also collected demographic data including participants’ age at the time of survey completion (in years), gender, gravidity (e.g., whether the participant’s most recent pregnancy was their first pregnancy (Costas et al. 2014)), plurality (the number of fetuses carried), mode of conception (e.g., unassisted, or through assisted reproductive technology), pre-conception body-mass index (calculated from participants’ self-reported height and estimated body mass at the time of conception), region of residence (e.g., Australia or Europe), marital status (e.g., single or married), highest level of education achieved (e.g., Bachelor’s degree), and competitive sport participation in the six months prior to conception. The first trimester was defined as gestational weeks 0 to 14, the second trimester was defined was weeks 15 to 28, and the third trimester was defined as week 29 onwards (Kubler et al. 2024).

### Data sources

Participants were provided with written and visual instructions for downloading and sharing the exercise data recorded on their fitness apps or wearable devices during the 12 weeks prior to conception, pregnancy, and the first 12 weeks postpartum. Due to the retrospective nature of the study, we were unable to provide participants with instructions for the setup or calibration of their personal wearable devices. The data for all additional outcomes were self-reported by participants through online surveys. The exercise and survey data were collected and managed using Research Electronic Data Capture (REDCap) tools hosted at Australian Catholic University (Harris et al. 2009, 2019; Johns Hopkins University, 2022; Lawrence et al. 2020; Wilson 2021).

### Study size

A sample size was estimated for a mixed model of repeated measures with an exchangeable correlation structure. Weekly exercise duration was used as the primary outcome because it is a core component of exercise behaviour, and previous literature provided sufficient data to inform model assumptions (Darroch et al. 2022). The sample size calculation suggested that 63 participants were needed to reject the null hypothesis that the difference in exercise duration between trimesters was zero with probability (power) 0.80. The Type I error probability associated with this test of the null hypothesis was 0.05.

### Data cleaning

On weeks where a participant did not record any exercise sessions, we set the weekly exercise duration and frequency as zero. This conservative approach has been adopted in previous research investigating exercise behaviours using training diaries (Donkers et al. 2020). One participant’s data was removed from the pre-conception and postpartum analyses, as exercise data from more than 66% of the weeks in these periods were not provided. Exercise sessions with recorded durations (> 600 min) or HR values (< 40% HR_max_) that were deemed to be implausible were also removed prior to data analysis. The excluded data were reviewed by RDJ and KLO prior to being removed.

### Statistical methods

Descriptive statistics were calculated for all outcomes to provide context for subsequent analyses (see Supplementary Tables 1–5, Online Resource 1).

Changes in participants’ exercise behaviours over time were assessed using mixed-effects models. We conducted between-phase comparisons using generalised linear mixed models (GLMMs) with: a Tweedie distribution and log link for weekly exercise duration (minutes·wk^− 1^) and aerobic exercise volume (MET-min·wk^− 1^); a Gamma distribution for mean exercise session duration (minutes·session^− 1^); and a Poisson distribution for weekly exercise frequency (sessions·wk^− 1^). A linear mixed-effects model (LMM) was used to assess between-phase changes in mean aerobic exercise intensity (% age-predicted HR_max_). The models for each outcome included phase as a fixed effect, and a random intercept and random slope for period grouped by participant identification number, allowing for individual variation in baseline exercise behaviours as well as changes in exercise characteristics throughout the study period. Within-phase changes in all outcomes were modelled using the same family-distribution combinations, with week as the fixed effect. The models were built using the *glmmTMB* (version 1.1.10) (Brooks et al. 2024) and *lme4* (version 1.1.35.5) (Bates et al. 2017) packages and *glmmTMB*, *glmer*, and *lmer* functions in R (version 4.4.2) (R Core Team 2022). To ensure model assumptions were met, the *check_model* function from the *performance* package (version 0.12.4) (Lüdecke et al. 2025) was used, which assesses multicollinearity, heteroscedasticity, influential observations, and the linearity of observed relationships. Multiple helper functions were used to clean and analyse the study data (Attali and Baker 2023; Barrett et al. 2024; Bates et al. 2024; Ben-Shachar et al. 2024; Bolker et al. 2024; Christensen 2024; Team et al. 2025; Dunn 2022; Fox et al. 2024; Gohel et al. 2024; Kuznetsova et al. 2020; Loy et al. 2023; Ooms et al. 2024; Ooms and McNamara 2024; Schauberger et al. 2024; Sievert et al. 2024; Smyth et al. 2023; Wickham et al. 2019, 2023; Wickham and Bryan 2023; Wilke 2024).

Wald chi-square tests were used to examine the effect of study phase (e.g., pre-conception, pregnancy, and postpartum) on exercise duration, frequency, intensity, and volume. Post-hoc pairwise comparisons between periods were conducted using the Tukey method for multiple comparisons with statistical significance set at α = 0.05, as implemented in the *emmeans* package (version 1.10.6) (Russell V. Lenth et al. 2024) in R (R Core Team, 2022). Beta coefficients were used to quantify the magnitude of the effect of each additional week (during the pre-conception, pregnancy, and postpartum phases) on exercise behaviours (e.g., exercise duration, frequency, intensity, and volume). Results are presented as percentage differences with 95% confidence intervals and *p*-values, derived from model estimates.

## Results

### Participants

**Fig. 1:**
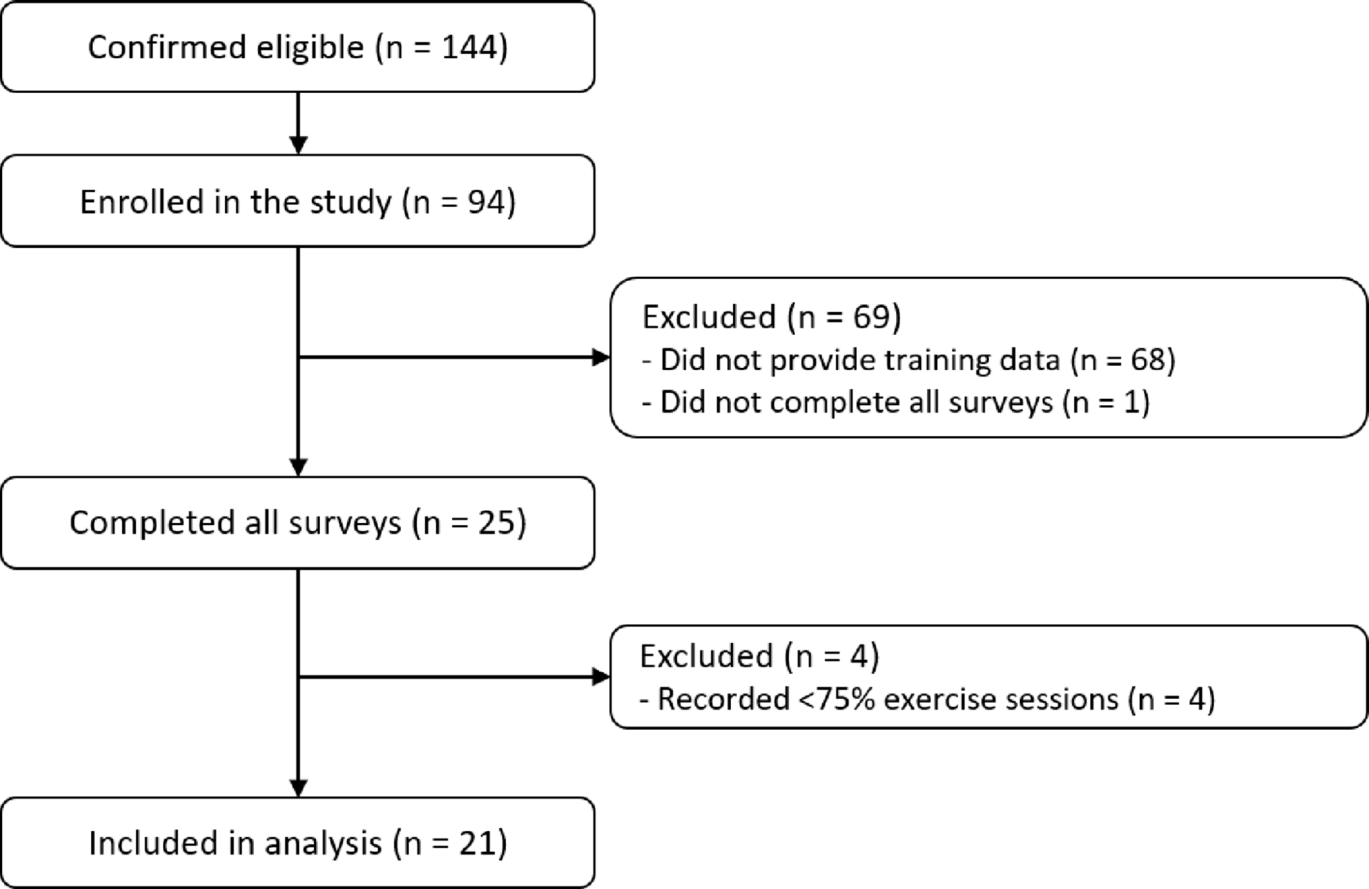
STROBE flow diagram of eligible participants for the present study

Data from 21 participants were included in the analysis. While 94 participants enrolled in the study, 68 participants did not upload sufficient training data, four participants reported having recorded fewer than 75% of the exercise sessions completed during pregnancy, and one participant did not complete the study surveys. The numbers of participants included in each stage of the study, as well as reasons for exclusion in each stage, are provided in Fig. 1.

### Participant characteristics

The participants in the study population were typically primiparous (i.e., their most recent pregnancy was their first pregnancy), highly educated, married, and located in North or Central America (Table 1). All participants had singleton pregnancies and described their gender as ‘woman or female’. Participants were aged 26 to 41 years at the time of survey completion and had a median pre-conception body mass index (BMI) of 22.6 kg.m^− 2^. Three participants reported having used assisted reproductive technology. Eight participants reported having competed in sport during the six months pre-conception, with the remaining 13 participants meeting the self-reported weekly exercise participation inclusion criteria. Of the participants who competed in sport during the pre-conception period, most reported that their highest ever level of sporting competition had been at a fun run or charity event, or at the local level (e.g., Parkrun or social sport).

**Table 1:** Characteristics of included participants

Characteristics	Overall (*n* = 21)		
	*n* (%)	Mean (SD)	Median [Min, Max]
Age at survey completion (y)	-	35 (4)	34 [26, 41]
Gender	-	-	-
*Woman or female*	21 (100)	-	-
First pregnancy	12 (57.1)	-	-
Singleton pregnancy	21 (100)	-	-
Mode of conception	-	-	-
*Unassisted*	18 (87.5)	-	-
*Assisted reproductive technology*	3 (14.3)	-	-
Pre-conception BMI	-	23.3 (3.0)	22.6 [19.7, 31.6]
Region of residence	-	-	-
*Australia*	7 (33.3)	-	-
*Europe*	1 (4.8)	-	-
*North or Central America*	13 (61.9)	-	-
Marital status	-	-	-
*Single*	1 (4.8)	-	-
*Married*	20 (95.2)	-	-
Highest level of education	-	-	-
*Bachelor’s degree*	7 (33.3)	-	-
*Graduate diploma*	1 (4.8)	-	-
*Master’s degree*	7 (33.3)	-	-
*Doctoral degree*	6 (28.6)	-	-
Competed in sport in the six months pre-conception	9 (42.9)	-	-
Main competitive sport(s) ^*b*^	-	-	-
*CrossFit*	1 (4.8)	-	-
*Netball*	1 (4.8)	-	-
*Powerlifting*	1 (4.8)	-	-
*Running*	5 (23.8)	-	-
*Swimming*	1 (4.8)	-	-
*Not applicable*	13 (61.9)	-	-
Highest ever level of competition ^*b*^	-	-	-
*Fun run or charity event*	4 (19.0)	-	-
*Local level*	2 (9.5)	-	-
*National level*	1 (4.8)	-	-
*Other*^*a*^	1 (4.8)	-	-
Not applicable	13 (61.9)	-	-

BMI, Body mass index

^a^‘Other’ highest ever level of competitive sport: age-group Ironman; ^b^ Responses from participants who reported competing in sport in the six months pre-conception (*n* = 8);

### Weekly exercise duration

There was a significant main effect of phase (pre-conception, pregnancy, or postpartum) on weekly exercise duration (χ^2^ (2) = 41.25, *p* < 0.001). Post-hoc pairwise comparisons indicated that weekly exercise duration in the pre-conception period was 17.6% higher (95% CI [0.6%, 37.5%]) than during pregnancy, and 80.5% higher (95% CI [41.7%, 129.8%]) than during the first 12 weeks postpartum (Table 2). Weekly exercise duration was 53.4% higher (95% CI [13.8%, 106.8%]) during pregnancy than in the postpartum period.

**Table 2:** Post-hoc comparisons of weekly exercise duration between phases

Comparison	Estimate	SE	95% CI Lower	95% CI Upper	*p*
Pre-conception vs. Pregnant	1.176	0.067	1.006	1.375	0.040
Pre-conception vs. Postpartum	1.805	0.103	1.417	2.298	<0.001
Pregnant vs. Postpartum	1.534	0.127	1.138	2.068	0.002

*SE*, Standard error; *CI*, Confidence interval

**Fig. 2:**
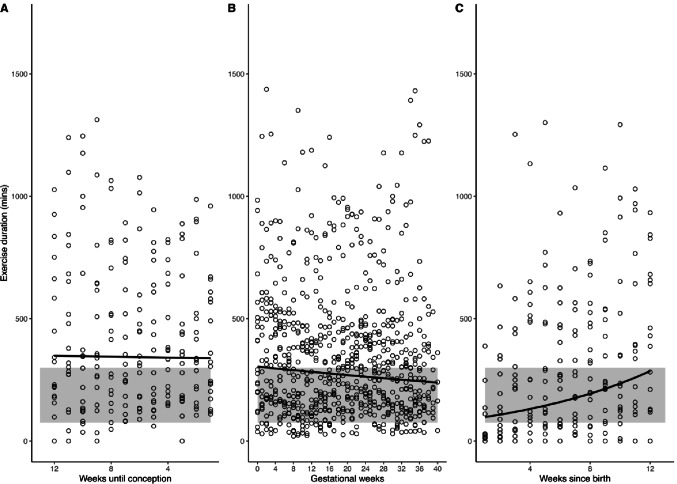
Estimated weekly exercise duration (min·wk^− 1^) in the pre-conception (**A**), pregnancy (**B**) and postpartum (**C**) phases from the mixed effects models, alongside the original data points. The grey bands illustrate the weekly exercise duration range of moderate to vigorous exercise (75 to 300 min·week^− 1^), commonly reported to be safe and beneficial during pregnancy (Claiborne et al. 2023)

Participants’ baseline weekly exercise duration 12 weeks prior to conception was 337 min·wk^− 1^ (95% CI [248, 451]). Participants’ weekly exercise duration was 304 min·wk^− 1^ (95% CI [223, 413]) in the first week of pregnancy (Table 3). There were no significant changes in participants’ weekly exercise duration throughout pre-conception or pregnancy. Participants’ estimated weekly exercise duration was 88 min·wk^− 1^ (95% CI [44, 168]) in the first week postpartum, and increased by 10.2% (95% CI [4.4%, 16.6%]) with each additional week postpartum. The trends in participants’ weekly exercise durations across each study phase are shown in Fig. 2.

**Table 3:** Generalised linear mixed model estimates of weekly exercise duration trends in the pre-conception, pregnancy, and postpartum phases

Phase	Predictor	Estimate	95% CI Lower	95% CI Upper	% Change
Pre-conception	Intercept	337.812	248.439	451.245	
	Weeks pre-conception	1.003	0.984	1.025	+0.259
Pregnancy	Intercept	304.211	222.917	412.736	
	Gestational weeks	0.994	0.987	1.002	-0.604
Postpartum	Intercept	88.939	43.945	168.102	
	Weeks postpartum	1.102	1.044	1.166	+10.213

*CI*, Confidence interval

Nineteen participants (90%) exceeded current guideline recommendations for weekly exercise duration (i.e., exercised for > 300 min·wk^− 1^) in at least one week during pregnancy. Twelve participants (57%) exceeded weekly exercise duration recommendations in at least 10 weeks while pregnant.

### Exercise session duration

**Fig. 3:**
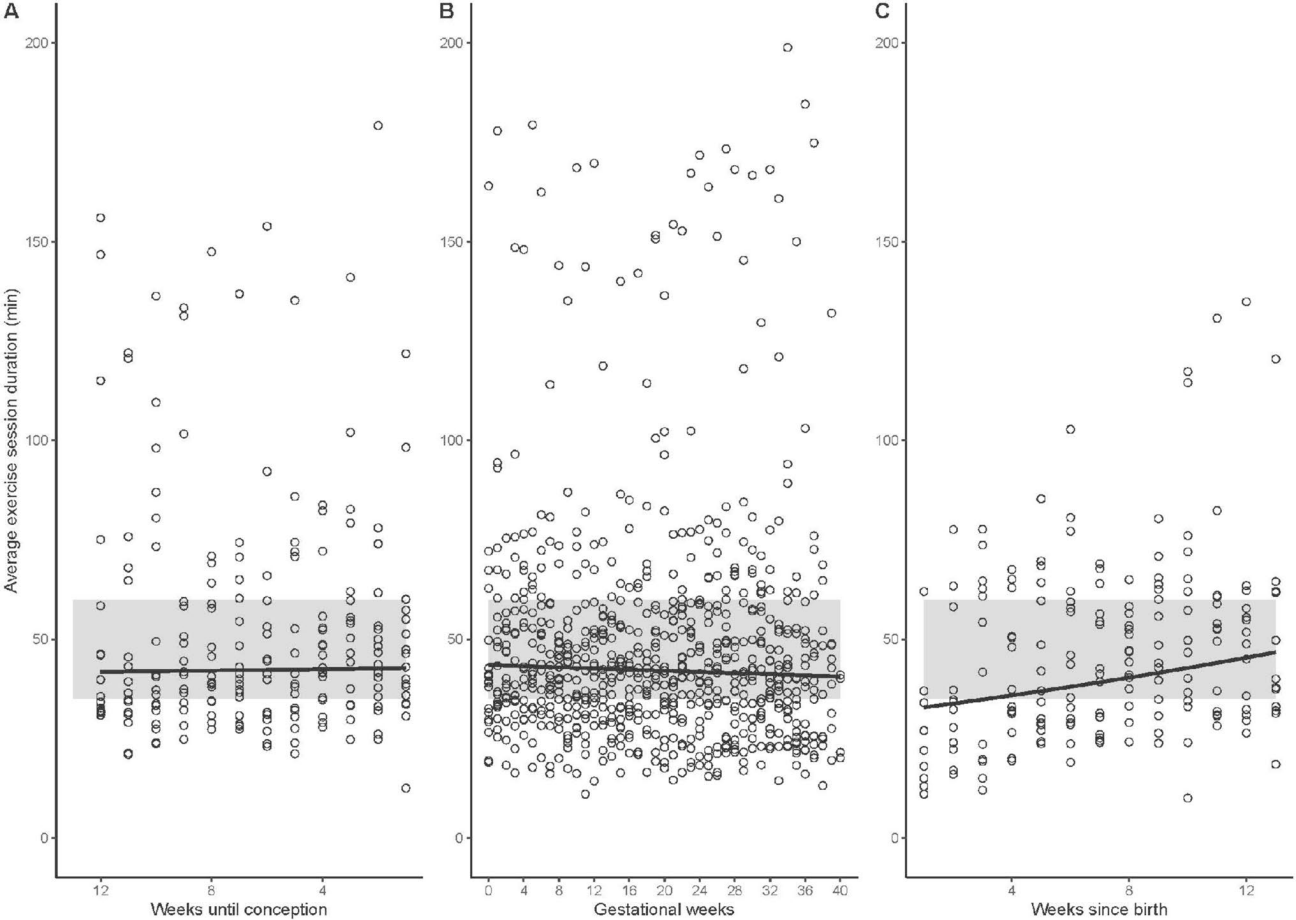
Estimated exercise session duration (min·session^− 1^) in the pre-conception (**A**), pregnancy (**B**) and postpartum (**C**) phases from the mixed effects models, alongside the original data points. The grey bands illustrate the session duration range (35 to 60 min·session^− 1^) commonly reported to be safe and beneficial during pregnancy (Claiborne et al. 2023)

Study phase did not have a significant effect on participants’ average exercise session duration (χ^2^ (2) = 1.737, *p* = 0.420). Participants’ baseline exercise session duration 12 weeks prior to conception was 43 min·session^− 1^ (95% CI [35, 52]). Participants’ average exercise session duration was 44 min·session^− 1^ (95% CI [38, 50]) in the first week of pregnancy (Table 4). There were no significant changes in participants’ average exercise session duration with each additional week in the pre-conception or pregnancy phases. Participants’ average exercise session duration in the first week postpartum was 32 min·session^− 1^ (95% CI [27, 38]). The average exercise session duration increased by 3.0% (95% CI [1.6%, 4.4%]) with each additional week postpartum. The trends in participants’ average exercise session durations across each study phase are provided in Fig. 3.

**Table 4:** Generalised linear mixed model estimates of exercise session duration trends in the pre-conception, pregnancy, and postpartum phases

Phase	Predictor	Estimate	95% CI Lower	95% CI Upper	% Change
Pre-conception	Intercept	42.792	34.794	52.355	
	Weeks pre-conception	0.998	0.988	1.008	-0.002
Pregnancy	Intercept	43.518	37.530	50.471	
	Gestational weeks	0.998	0.997	1.000	-0.171
Postpartum	Intercept	31.882	26.828	37.799	
	Weeks postpartum	1.030	1.016	1.044	+2.982

*CI*, Confidence interval

Fifteen participants (71%) reported an average exercise session duration that exceeded current recommendations (> 60 min·session^− 1^) in at least one week during pregnancy. Four participants (19%) reported a weekly average exercise session duration of > 60 min·session^− 1^ in at least 10 weeks during pregnancy.

### Exercise frequency

There was a significant main effect of phase on weekly exercise frequency (χ^2^ (2) = 15.443, *p* < 0.001). Post-hoc pairwise comparisons indicated that weekly exercise frequency was 70.4% higher (95% CI [24.0%, 134.1%])  in the pre-conception period than during the postpartum period, and 55.2% higher (95% CI [15.3%, 108.9%]) in pregnancy than in postpartum (Table 5). There was no significant difference in weekly exercise frequency between the pre-conception and pregnancy phases.

**Table 5:** Post-hoc comparisons of weekly exercise frequency between phases

Comparison	Estimate	SE	95% CI Lower	95% CI Upper	*p*
Pre-conception vs. Pregnant	1.098	0.063	0.947	1.273	0.301
Pre-conception vs. Postpartum	1.704	0.136	1.240	2.341	<0.001
Pregnant vs. Postpartum	1.552	0.127	1.153	2.089	0.002

*SE*, Standard error; *CI*, Confidence interval

**Fig. 4:**
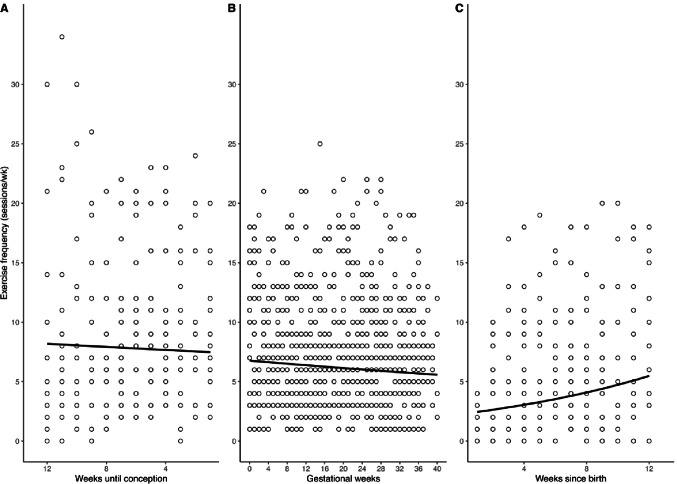
Estimated weekly exercise frequency (sessions·wk^− 1^) in the pre-conception (**A**), pregnancy (**B**) and postpartum (**C**) phases from the mixed effects models, alongside the original data points

Baseline weekly exercise frequency 12 weeks prior to conception was seven sessions per week (95% CI [5, 10]), which did not change significantly throughout pre-conception or pregnancy (Table 6). In the first week postpartum, the average participant completed two exercise sessions. Exercise frequency then increased by 7.6% (95% CI [1.8%, 13.9%]) with each additional week postpartum. The trends in participants’ reported weekly exercise frequencies across each study phase are shown in Fig. 4.

### Aerobic exercise intensity

The heart rate monitoring devices used by participants throughout pregnancy are provided in Supplementary Table 6 (Online Resource 2). The most commonly used devices were Garmin models (*n* = 9), followed by Apple Watch (*n* = 4), Fitbit (*n* = 2), WHOOP (*n* = 2), and Withings (*n* = 1) devices. Heart rate data were not available for three participants, who were subsequently excluded from the aerobic exercise intensity and volume analyses. Three participants (16%) included in the exercise intensity and volume analyses experienced hypothyroidism in the six months prior to conception.

**Table 6:** Generalised linear mixed model estimates of weekly exercise frequency trends in the pre-conception, pregnancy, and postpartum phases

Phase	Predictor	Estimate	95% CI Lower	95% CI Upper	% Change
Pre-conception	Intercept	7.417	5.402	9.967	
	Weeks pre-conception	1.008	0.988	1.032	+0.823
Pregnancy	Intercept	6.765	5.129	8.855	
	Gestational weeks	0.995	0.987	1.003	-0.482
Postpartum	Intercept	2.277	1.222	4.026	
	Weeks postpartum	1.076	1.018	1.139	+7.590

*CI*, Confidence interval

There was a significant main effect of phase on weekly average aerobic exercise intensity (χ^2^ (2) = 29.199, *p* < 0.001). Post-hoc pairwise comparisons indicated that aerobic exercise intensity was 8.0% higher (95% CI [3.9%, 12.2%]) during pre-conception than postpartum (Table 7). There were no significant differences in average aerobic exercise intensity between pre-conception and pregnancy, or between pregnancy and postpartum.

**Table 7:** Post-hoc comparisons of average weekly exercise intensity between phases

Comparison	Estimate	SE	95% CI Lower	95% CI Upper	*p*
Pre-conception vs. Pregnant	1.011	0.015	0.974	1.050	0.730
Pre-conception vs. Postpartum	1.080	0.015	1.039	1.122	<0.001
Pregnant vs. Postpartum	1.068	0.019	1.016	1.122	0.010

*SE*, Standard error; *CI*, Confidence interval

**Fig. 5:**
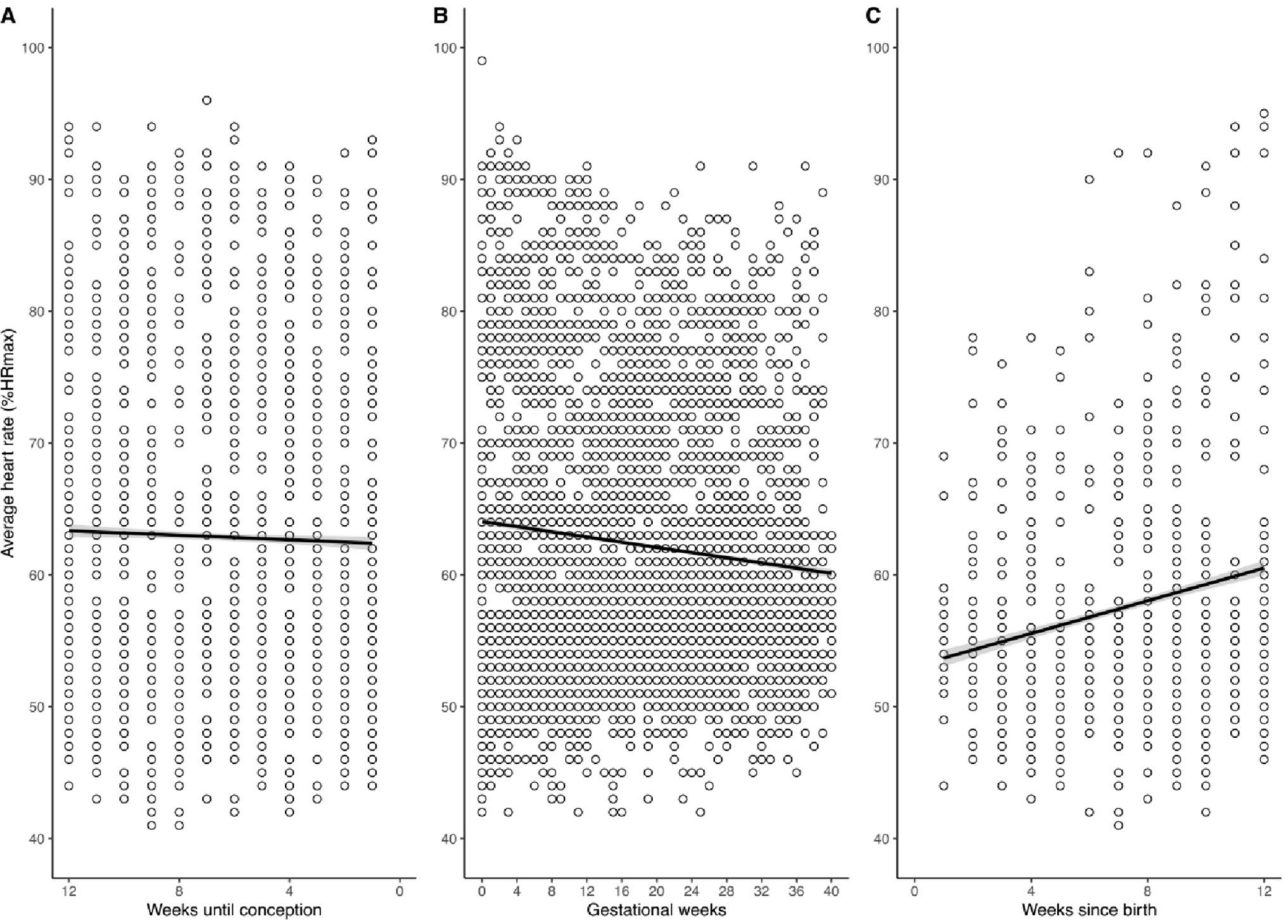
Estimated weekly average exercise intensity (%HR_max_) in the pre-conception (**A**), pregnancy (**B**) and postpartum (**C**) phases from the mixed effects models, alongside the original data points

Average aerobic exercise intensity 12 weeks prior to conception was 63% age-predicted HR_max_ (95% CI [60%, 68%]) in each session, with no significant change with each additional week throughout the pre-conception phase (Table 8). In the first week of pregnancy, average aerobic exercise intensity was 64% age-predicted HR_max_ (95% CI [61%, 68%]) in each session, which did not change significantly with each additional gestational week. In the first week postpartum, participants’ average aerobic exercise intensity was 55.6% age-predicted HR_max_ (95% CI [52%, 59%]) in each session, which increased by 0.9% (95% CI [0.6%, 1.1%]) with each additional week postpartum. The trends in participants’ average aerobic exercise intensity across each phase are shown in Fig. 5.

**Table 8:** Generalised linear mixed model estimates of average weekly exercise intensity trends in the pre-conception, pregnancy, and postpartum phases

Phase	Predictor	Estimate	95% CI Lower	95% CI Upper	% Change
Pre-conception	Intercept	63.411	59.512	67.566	
	Weeks pre-conception	0.998	0.995	1.001	-0.201
Pregnancy	Intercept	64.362	61.298	67.580	
	Gestational weeks	0.999	0.999	1.000	-0.087
Postpartum	Intercept	55.610	52.197	59.245	
	Weeks postpartum	1.009	1.006	1.011	0.852

*CI*, Confidence interval

Light-intensity, 40 < 55% HR_max_; Moderate-intensity, 55 < 70% HR_max_; Vigorous-intensity, 70 < 90% HR_max_; High-intensity, ≥ 90% HR_max_

Five participants (28%) completed aerobic exercise sessions where their average HR was within the high-intensity range (> 90% age-predicted HR_max_) at least once during pregnancy. Three participants (17%) completed at least 10 high-intensity aerobic exercise sessions (average HR > 90% age-predicted HR_max_) during pregnancy. Characteristics and birth outcomes for the participants who engaged in high-intensity exercise are provided in Supplementary Tables 7 and 8 (Online Resource 3).

### Aerobic exercise volume

There was a significant main effect of phase on weekly aerobic exercise volume (χ^2^ (2) = 33.267, *p* < 0.001). Post-hoc pairwise comparisons indicated that aerobic exercise volume was 205.1% higher (95% CI [92.5%, 383.6%]) during pre-conception than in postpartum, and 118.9% higher (95% CI [29.5%, 270.3%]) during pregnancy than in the postpartum period (Table 9). There was no significant difference in weekly aerobic exercise volume between the pre-conception and pregnancy phases.

**Table 9:** Post-hoc comparisons of average weekly exercise volume between phases

Comparison	Estimate	SE	95% CI Lower	95% CI Upper	*p*
Pre-conception vs. Pregnant	1.394	0.155	0.970	2.003	0.081
Pre-conception vs. Postpartum	3.051	0.196	1.925	4.836	<0.001
Pregnant vs. Postpartum	2.189	0.224	1.295	3.703	0.001

*SE*, Standard error; *CI*, Confidence interval

**Fig. 6:**
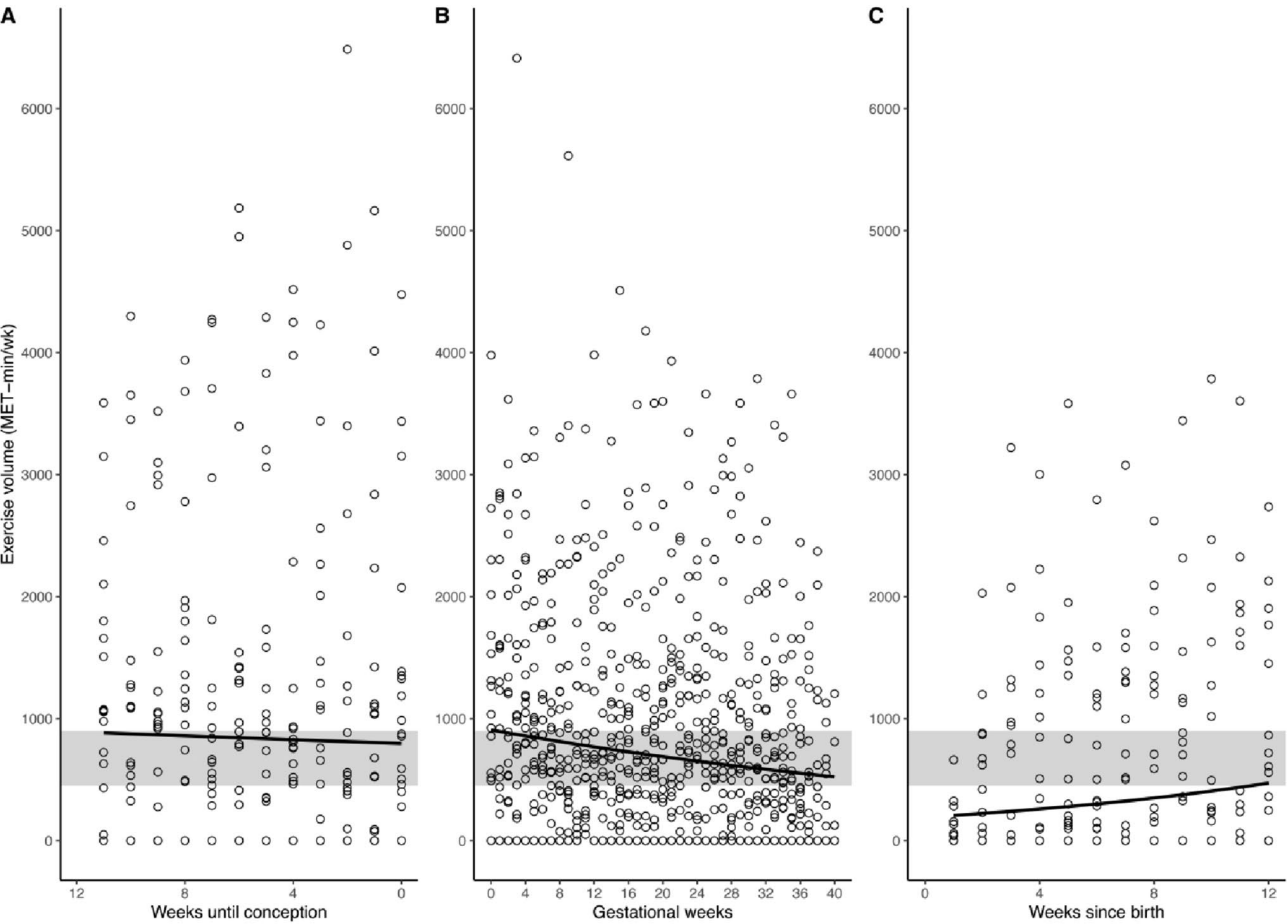
Estimated weekly aerobic exercise volume (MET-min·wk^− 1^) in the pre-conception (**A**), pregnancy (**B**) and postpartum (**C**) phases from the mixed effects models, alongside the original data points. The grey bands illustrate the weekly recommendations included in the 2021 Australian Guidelines for Physical Activity during Pregnancy and Postpartum (equivalent to 450 to 900 MET-min·wk^− 1^) (Brown et al. 2022)

Participants’ baseline weekly aerobic exercise volume 12 weeks prior to conception was 892 MET-min·wk^− 1^ (95% CI [485, 1589]), which did not change with each additional week pre-conception (Table 10). In the first week of pregnancy, participants’ average aerobic exercise volume was 907 MET-min·wk^− 1^ (95% CI [619, 1322]), which decreased by 1.4% (95% CI [1.0%, 1.7%) with each gestational week. In the first week postpartum, participants’ average aerobic exercise volume was 192 MET-min·wk^− 1^ (95% CI [90, 396]), which increased by 7.8% (95% CI [4.2%, 11.5%]) with each additional week postpartum. The trends in participants’ weekly aerobic exercise volume across each phase are shown in Fig. 6.

**Table 10:** Generalised linear mixed model estimates of weekly aerobic exercise volume trends in the pre-conception, pregnancy, and postpartum phases

Phase	Predictor	Estimate	95% CI Lower	95% CI Upper	% Change
Pre-conception	Intercept	892.486	485.008	1589.063	
	Weeks pre-conception	0.991	0.973	1.009	-0.946
Pregnant	Intercept	906.737	619.395	1322.505	
	Gestational weeks	0.986	0.983	0.990	-1.366
Postpartum	Intercept	192.256	89.883	396.340	
	Weeks postpartum	1.078	1.042	1.115	+7.759

*CI*, Confidence interval

Seventeen participants (94% of the 18 participants who reported the intensity of their sessions) exceeded existing weekly exercise volume recommendations (> 900 MET-min·wk^− 1^) on at least one week during pregnancy. Ten participants (56%) completed more than 900 MET-min of exercise on at least ten weeks during pregnancy.

### Exercise modality

The most common exercise modality across all phases was walking (Table 11). Running was the second most-common aerobic exercise modality during pre-conception and the first trimester, became less frequent in the second and third trimesters, and then increased in the first 12 weeks postpartum. Some participants maintained engagement in diverse aerobic exercise modalities such as bushwalking, rowing, and stand-up paddleboarding during pregnancy, but only one bushwalking session was reported in the postpartum period. Engagement in resistance training increased slightly from pre-conception to pregnancy, remained consistent across all three trimesters, and then decreased in the postpartum period. Participation in Pilates peaked in the second trimester of pregnancy. One participant engaged in closed-circuit rebreather and single-gas diving prior to conception and early in the first trimester, but discontinued diving after the third week of pregnancy.

**Table 11:** Number of self-reported exercise sessions completed for each exercise modality

Modality	Pre-conception *n* (%)	Trimester 1 *n* (%)	Trimester 2 *n* (%)	Trimester 3 *n* (%)	Postpartum *n* (%)
**Aerobic**	**1505 (78.3)**	**1511 (73.9)**	**1473 (74.7)**	**1218 (72.8)**	**1032 (76.4)**
Walking	786 (52.2)	772 (51.1)	818 (55.5)	768 (63.1)	773 (74.9)
Running	311 (20.7)	336 (22.2)	169 (11.5)	83 (6.8)	114 (11)
Cycling	317 (21.1)	288 (19.1)	323 (21.9)	254 (20.9)	102 (9.9)
Swimming	21 (1.4)	11 (0.7)	15 (1.0)	43 (3.5)	16 (1.6)
Other aerobic	23 (1.5) ^a^	33 (2.2) ^b^	38 (2.6) ^c^	30 (2.5) ^d^	1 (0.1) ^e^
Unspecified	47 (3.1)	71 (4.7)	110 (7.5)	40 (3.3)	26 (2.5)
**Resistance**	**222 (11.5)**	**282 (13.8)**	**308 (15.6)**	**234 (14.0)**	**122 (9.0)**
Pilates	26 (1.4)	33 (1.6)	44 (2.2)	10 (0.6)	2 (0.1)
Weightlifting	-	1 (0.4)	18 (6.8)	22 (9.8)	4 (3.3)
Powerlifting	2 (1)	-	13 (4.9)	7 (3.1)	-
Barre	1 (0.5)	1 (0.4)	-	-	-
Unspecified	193 (92.3)	247 (99.2)	233 (88.3)	195 (87.1)	116 (96.7)
**Yoga**	**75 (3.9)**	**89 (4.4)**	**72 (3.7)**	**89 (5.3)**	**76 (5.6)**
**Other**	**9 (0.5)** ^**f**^	**12 (0.6)** ^**g**^	**-**	**-**	**-**
**Unspecified**	**112 (5.8)**	**151 (7.4)**	**118 (6.0)**	**133 (7.9)**	**121 (9.0)**
**Total**	**1923 (100)**	**2045 (100)**	**1971 (100)**	**1674 (100)**	**1351 (100)**

^a^‘Other aerobic’ activities in the pre-conception period were bushwalking/hiking (*n* = 16), rowing (*n* = 2), kayaking (*n* = 2), mountain biking (*n* = 1), stair stepper (*n* = 1), and stand up paddleboarding (*n* = 1)

^b^‘Other aerobic’ activities in trimester one were bushwalking/hiking (*n* = 22), stair stepper (*n* = 4), rowing (*n* = 3), elliptical (*n* = 2), stand up paddleboarding (*n* = 1), and paddle sports (*n* = 1)

^c^‘Other aerobic’ activities in trimester two were elliptical (*n* = 23), stair stepper (*n* = 7), bushwalking/hiking (*n* = 6), Nordic skiing (*n* = 1), and stand up paddleboarding (*n* = 1)

^d^‘Other aerobic’ activities reported in trimester three were stair stepper (*n* = 13), elliptical (*n* = 11), stand up paddleboarding (*n* = 3), rowing (*n* = 2), bushwalking/hiking (*n* = 1)

^e^‘Other aerobic’ activity in the postpartum period was bushwalking/hiking (*n* = 1)

^f^‘Other’ activities in the pre-conception period were closed-circuit rebreather diving (*n* = 9)

^g^‘Other’ activities in trimester one were basketball (*n* = 4), closed-circuit rebreather diving (*n* = 3), single-gas diving (*n* = 2), rock climbing (*n* = 2), and racquetball (*n* = 1)

### Birth and pregnancy outcomes

The most commonly reported health condition during pregnancy was urinary incontinence, followed by diastasis recti abdominus (Table 12). Median gestational weight gain was 14.3 kg (IQR = 8.02). When participants’ reported gestational weight gain was compared to the BMI-specific Institute of Medicine recommendations (Institute of Medicine, 2009), one participant’s weight gain was within the recommended range, three participants gained an insufficient amount of weight, and sixteen participants’ weight gain was categorised as ‘excessive’.

All infants were within the normal birthweight range (2500 to 4500 g) (Table 13) (*The International statistical classification of diseases and related health problems*,* 10th revision*,* Australian modification (ICD-10-CM)*, 2002; Royal Australian and New Zealand College of Obstetricians and Gynaecologists, 2021). Most infants were born at full term (*n* = 14), while five were at early term (37 to 38 weeks (American College of Obstetricians and Gynecologists 2013) and two were late preterm (34 to 36 weeks (*Mosby’s dictionary of medicine*,* nursing & health professions*, 2014). The most common birth complications were perineal tears (*n* = 8), followed by fetal distress (*n* = 4) and meconium staining (*n* = 3).

**Table 12:** Pregnancy-related conditions and outcomes

Outcomes	Overall (*n*=21)		
	*n* (%)	Mean (SD)	Median [IQR]
Intrauterine growth restriction	1 (4.8)	-	-
Gestational diabetes mellitus	2 (9.5)	-	-
Pre-eclampsia	2 (9.5)	-	-
Urinary incontinence	12 (57.1)	-	-
Faecal incontinence	1 (4.8)	-	-
Hyperemesis gravidarum	1 (4.8)	-	-
Pelvic organ prolapse ^a^	1 (4.8)	-	-
Placenta praevia	2 (9.5)	-	-
Diastasis recti abdominus	8 (38.1)	-	-
Pre-conception BMI (kg·m^-2^) ^b^	-	23.28 (3.0)	22.6 [2.9]
‘Healthy weight’	16 (76.2)	-	-
Overweight	3 (14.3)	-	-
Obese	1 (4.8)	-	-
Not specified	1 (4.8)	-	-
Gestational weight gain (kg) ^c^	-	14.64 (5.9)	14.30 [8.0]
Insufficient	3 (14.3)	-	-
Adequate	1 (4.8)	-	-
Excessive	16 (76.2)	-	-
Not specified	1 (4.8)	-	-

*BMI*, Body mass index; *kg*, Kilograms

^a^Stage 1 bladder prolapse (*n* = 1)

^b^Categories based on the Australian Government guidance on BMI and waist measurement [82]

^c^Categories based on the Institute of Medicine BMI-specific guidelines for gestational weight gain [78]

**Table 13:** Birth outcomes

Outcomes	Overall (*n*=21)		
	*n* (%)	Mean (SD)	Median [IQR]
Infant sex	-	-	-
*Female*	8 (38.1)	-	-
*Male*	13 (61.9)	-	-
Birth weight (g)	-	3333 (464.2)	3359 [513.7]
*Within normal limits (2500–4500 g)*	21 (100)	-	-
Gestational age at birth (wk)	-	38.8 (1.6)	39.0 [2.0]
*Late preterm (34 - 36 wk)*	2 (9.5)	-	-
*Early term (37 - 38 wk)*	5 (23.8)	-	-
*Full term (39 - 41 wk)*	14 (66.7)	-	-
*Post-term (≥ 42 wk)*	0 (0)	-	-
Mode of delivery	-	-	-
*Vaginal*	16 (76.2)	-	-
*Caesarean section (planned)*	2 (9.5)	-	-
*Caesarean section (urgent)* ^a^	1 (4.8)	-	-
*Caesarean section (emergency)* ^b^	2 (9.5)	-	-
Delivery interventions	-	-	-
*Epidural*	11 (52.4)	-	-
*Induction*	7 (33.3)	-	-
*Episiotomy*	2 (9.5)	-	-
*Forceps*	1 (4.8)	-	-
*Vacuum*	1 (4.8)	-	-
*No intervention*	4 (19.0)	-	-
Birth complications	-	-	-
*Perineal tear* ^c^	8 (38.1)	-	-
*Fetal distress*	4 (19.0)	-	-
*Meconium staining*	3 (14.2)	-	-
*Prolonged labour*	2 (9.5)	-	-
*Stalled labour*	2 (9.5)	-	-
*Umbilical cord complications*	1 (4.8)	-	-
*Excessive bleeding*	1 (4.8)	-	-
*Breech birth*	1 (4.8)	-	-
*Shoulder dystocia*	0 (0)	-	-
*Perinatal asphyxia*	0 (0)	-	-
*Other* ^e^	1 (4.8)	-	-
*None of the above*	3 (14.2)	-	-

*wk*, Weeks

^a^Urgent caesarean section due to membrane rupture prior to date of planned caesarean section

^b^Emergency caesarean sections due to stalled labour (*n* = 1) and obstructed labour (*n* = 1)

^c^First-degree tear *n* = 1; Second-degree tear *n* = 6; Third-degree tear *n* = 1

^d^‘Other’ reported complication was uterine mass prolapse during birth (*n* = 1)

## Discussion

The aim of this study was to characterise how recreationally active individuals modified their exercise behaviours from 12 weeks prior to conception, through pregnancy, and in the first 12 weeks postpartum. The results suggest that some active individuals (*n* = 19) exercise at levels that exceed current weekly exercise duration recommendations (Brown et al. 2022; Hayman et al. 2023) during pregnancy. Despite this, the observed decrease in aerobic exercise volume throughout pregnancy, as well as the progressive increase in exercise participation in the first 12 weeks postpartum, indicate that training load is still modified throughout the antenatal and postpartum periods. While walking was the most common exercise modality reported, we evidence that some individuals participate in diverse aerobic, resistance, and other sport-related activities during pregnancy. These findings highlight that some individuals who are not competitive athletes (McKay et al. 2022) remain highly active throughout their pregnancies, and may require individualised support to modify their training behaviours throughout gestation.

Our results suggest that high weekly durations of antenatal exercise are not exclusive to elite athletes. While previous research has established that some elite athletes maintain high training loads of up to 12.9 h per week during pregnancy (Sigurdardottir et al. 2019; Solli and Sandbakk 2018; Wowdzia et al. 2020), no participants in our study met the criteria for classification as ‘elite/international level’ sportspeople outlined by McKay and colleagues (McKay et al. 2022). Indeed, of the participants who competed in sport during the six months prior to conception, most reported that their highest ever level of competitive experience had been at the fun run/charity event or local levels. While it seems likely that participants’ abilities to tolerate higher volumes of antenatal exercise may be relative to their pre-conception exercise behaviours, two randomised controlled trials found that participants who did not engage in any regular physical activity prior to conception engaged in 150 to 180 min of vigorous-intensity aerobic exercise per week during pregnancy (i.e., meeting or exceeding the highest weekly recommendations included in current guidelines (Brown et al. 2022)) without experiencing a significant reduction in infant birthweight (Baciuk et al. 2008; Marquez-Sterling et al. 2000) or gestational age at birth (Baciuk et al. 2008). It is possible that participants’ abilities to maintain extended weekly exercise durations throughout gestation are more strongly influenced by the severity of symptoms such as nausea, fatigue, pelvic floor dysfunction, and musculoskeletal discomfort, or perceptions of the safety of continued exercise during pregnancy, than by pre-conception training behaviours (Baciuk et al. 2008; James et al. 2022; Marquez-Sterling et al. 2000). The extended weekly exercise durations reported by some participants highlight the need for further research into the safety of high-volume antenatal exercise to support active individuals in making informed decisions about their exercise participation during pregnancy.

Participants’ average exercise session duration remained between 30 and 60 min throughout pregnancy, aligning with international guideline recommendations (Rudin et al. 2021). While participants’ average session durations were within the recommended range, there was significant variation in reported session durations (range: 11 to 150 min). While exercise sessions lasting for 35 to 60 min are associated with improvements in maternal and neonatal health outcomes, there is a paucity of research investigating the health outcomes associated with consistently exercising in bouts lasting less than 35 min, or longer than 60 min (Claiborne et al. 2023). The Australian Institute of Sport recommend that pregnant athletes who wish to exercise for longer than 60 min at a time should seek advice from an appropriately qualified healthcare professional (Australian Institute of Sport 2025). Although the benefits of sessions < 35 min and the safety of those > 60 min remain unclear, our findings suggest that some pregnant individuals continue to engage in both. These results underscore the need for further research to inform clear guidance on the safety of extended exercise sessions during pregnancy.

Average aerobic exercise intensity was maintained within the moderate-to-vigorous range (55–89% HR_max_ (Norton et al. 2009) in the pre-conception, pregnancy, and postpartum periods, aligning with recommendations to exercise at < 90% HR_max_ during apparently healthy pregnancy (Beetham et al. 2019; Brown et al. 2022). While five participants completed at least one antenatal exercise session within the high-intensity range (≥ 90% HR_max_ (Norton et al. 2009), the number of reported high-intensity exercise sessions decreased substantially after the first trimester. The reduction in high-intensity exercise participation may reflect current uncertainty about the safety of high-intensity exercise intervals lasting for longer than one minute during pregnancy (Moholdt et al. 2024). It is important to interpret the exercise intensity data with caution, since intensities were determined based on participants’ age-predicted maximum heart rate values, and all exercise heart rate data was recorded with participants’ personal wearable devices (Dooley et al. 2017; Evenson and Hesketh 2023; Evenson and Spade 2020; Sarzynski et al. 2013). The available formulae for predicting maximal heart rate are associated with substantial errors of estimate (Evenson and Hesketh 2023). For example, the published standard error for the Fox formula is 12 bpm, suggesting that around 95% of participant’s estimated HR_max_ values will fall within ± 24 bpm of the true value (Evenson and Hesketh 2023). Additionally, the use of average HR to determine the intensity of each exercise session may result in the under-estimation of exposure to high-intensity exercise for participants who engaged in interval training sessions alternating between high and low-moderate intensity exercise bouts. Despite these potential limitations, our methods used for reporting exercise intensity are similar to previous studies using wrist-worn monitoring devices to investigate participants’ exercise characteristics throughout gestation (Bains et al. 2024). Future research involving monitoring pregnant individuals’ exercise behaviours using a standardised and validated device enabling minute-to-minute analysis of maternal HR may provide further insight into patterns of engagement in high-intensity exercise during gestation.

Participants engaged in different exercise modalities as pregnancy progressed. Participation in low-impact (Sigurdardottir et al. 2019) aerobic activities increased throughout pregnancy, with walking, swimming, cycling, and elliptical machine use making up progressively larger proportions of all exercise sessions completed in each trimester. In contrast, participation in running (a high-impact activity (Sigurdardottir et al. 2019)) decreased markedly, making up 22.2% of all aerobic exercise sessions completed in the first trimester, but only 6.8% in the third trimester. Further, resistance training made up a greater portion of exercise sessions completed in the second (15.6%) and third (14%) trimesters than in the pre-conception period (11.5%), while aerobic exercise participation was lower in the second (74.7%) and third (72.9%) trimesters than in the 12 weeks prior to conception (78.3%). The modification of exercise modality may have driven the observed reduction in weekly aerobic exercise volume throughout gestation. While identifying the reasons for these changes in exercise modality is beyond the scope of the current study, the finding that participants prioritised low-impact aerobic activity and resistance training as pregnancy progressed highlights the importance of offering diverse, adaptable exercise options in prenatal care settings.

### Limitations

This study has several limitations. Firstly, while it is important to consider participants’ exercise intensity distribution when investigating whether extended weekly exercise durations are safe during pregnancy, we were unable to analyse total weekly exercise volume (which considers weekly exercise duration *and* intensity) due to limitations in the available exercise intensity data. Our study presented detailed findings concerning participants’ weekly volumes and intensities of aerobic exercise, but we were unable to analyse resistance training participation in the same detail. Since our results suggested that resistance training made up a greater proportion of all sessions completed as pregnancy progressed, future studies should incorporate additional measures such as a Rating of Perceived Exertion (RPE) scale to enable monitoring of resistance training intensity and volume, and to facilitate the calculation of total weekly exercise volume.

The small sample size limits certainty in the results of the mixed effect models for each study phase. The small number of participants also precludes meaningful exploration of the associations between antenatal exercise behaviours and maternal and neonatal health outcomes. Many participants were excluded from the analysis after failing to share sufficient training data with the research team. While all participants were provided with written and visual instructions for downloading and sharing the training data recorded using common exercise monitoring platforms (e.g., Strava, Garmin, Fitbit, Apple Health), future studies should consider providing more comprehensive technical support to participants who are asked to share fitness app data. Due to the retrospective nature of the study, we were unable to control the setup and calibration of the wearable devices used by participants. We included participants who used any wearable device during pregnancy to maximise participation in the study, however a limitation of this approach is that we did not control the specific device models used. All available data about the devices and models used by participants have therefore been provided in Supplementary Table 6 (Online Resource 2).

While our study presented detailed findings concerning participants’ weekly volumes and intensities of aerobic exercise, we were unable to analyse resistance training participation in the same detail. Since our results suggested that resistance training made up a greater proportion of all sessions completed as pregnancy progressed, future studies should incorporate additional measures such as a Rating of Perceived Exertion (RPE) scale to enable monitoring of resistance training intensity and volume. Future prospective studies should also consider incorporating measures such as a 24-hour dietary recall or Food Frequency Questionnaire due to the potential confounding influences of dietary intake and hydration status on participants’ HR responses to exercise (Porto et al. 2023).

Participants’ pre-conception BMI values were used to categorise their gestational weight gain as being within the recommended range, ‘insufficient’, or ‘excessive’ based on the Institute of Medicine recommendations (Institute of Medicine, 2009). However, measures such as BMI may not provide accurate estimates of body composition and disease risk in all individuals (Wu et al. 2024). Future studies should consider additional methods for estimating pre-conception body composition (e.g., standardised measures such as dual-energy X-ray absorptiometry [DEXA], or estimations using equations such as the Navy Formula). Finally, individuals who had negative or traumatic experiences during their most recent pregnancy may have chosen not to enrol in this retrospective study, leading to potential under-reporting of adverse pregnancy and birth outcomes.

## Conclusions

This study highlights that some recreationally active individuals, who do not meet criteria for classification as athletes, exercise at volumes and intensities that exceed general guideline recommendations during pregnancy. Despite remaining highly active, participants progressively decreased their exercise participation throughout pregnancy before increasing again in the first 12 weeks postpartum, suggesting that training load was still modified as needed throughout the study period. These findings highlight the importance of providing individualised antenatal exercise guidance for people whose habitual activity levels exceed those suggested in antenatal exercise guidelines. Further research involving larger sample sizes may further improve our understanding of how recreationally active individuals modify their exercise behaviours throughout pregnancy and the postpartum period, as well as the associations between high levels of exercise participation and infant and maternal health outcomes.

## Supplementary Information

Below is the link to the electronic supplementary material.

Supplementary Material 1

Supplementary Material 2

Supplementary Material 3

## Data Availability

The re-identifiable datasets generated and analysed for this study are not publicly available to protect participant privacy. The processed data sets are available from the corresponding author upon reasonable request.

## Abbreviations

ACSM: American College of Sports Medicine
ACU: Australian Catholic University
App: Application
BMI: Body mass index
CI: Confidence interval
ESSA: Exercise and Sports Science Australia
GLMM: Generalised linear mixed-effects model
GWG: Gestational weight gain
HR_max_: Maximum heart rate
HR: Heart rate
HREC: Human research ethics committee
ID: Identification
IOM: Institute of Medicine
LMM: Linear mixed-effects model
MET: Metabolic equivalent
MET-min·wk^− 1^: Metabolic equivalent minutes per week
Min: Minutes
REDCap: Research Electronic Data Capture
SD: Standard deviation
STROBE: Strengthening the Reporting of Observational Studies in Epidemiology
Wk: Week

## Code availability

The R code used in this study is available from the corresponding author upon reasonable request.
