# Supplementary material for: Patterns in exercise behaviour across pregnancy: a retrospective cohort study of physically active individuals from pre-conception to postpartum
Source: Eur J Appl Physiol. 2026 Feb 25;126(6):3407–27. doi: 10.1007/s00421-026-06160-6 (PMC13287166; doi:10.1007/s00421-026-06160-6)
Supplement: Supplementary file 1 — Supplementary Material 1 [file 421_2026_6160_MOESM1_ESM.pdf]

Online Resource 1: Descriptive statistics for exercise behaviours across study phases

Article title: Patterns in exercise behaviour across pregnancy: a retrospective cohort study of physically active individuals from pre-conception to postpartum

Journal name: European Journal of Applied Physiology

Authors: Kate L Oxnard[1,2, 3], Rich D Johnston[1,2,4], Jemima G Spathis[1], Evelyn B Parr[5], Kassia S Beetham[1,2]

[1]School of Health and Behavioural Sciences, Australian Catholic University, 1100 Nudgee Road, Banyo, Brisbane, Queensland 4012, Australia

[2]Sports Performance, Recovery, Injury and New Technologies (SPRINT) Research Centre, Australian Catholic University, 1100 Nudgee Road, Banyo, Brisbane, Queensland, 4014, Australia

[3]College of Healthcare Sciences, James Cook University, 1 James Cook Drive, Douglas, Townsville, Queensland, 4814, Australia

[4]Carnegie Applied Rugby Research (CARR) Centre, Carnegie School of Sport, Leeds Beckett University, Leeds, United Kingdom

[5]Mary MacKillop Institute for Health Research, Australian Catholic University, Level 3, 250 Victoria Parade, Fitzroy, VIC 3065, Australia

Corresponding Author: Kassia Beetham

Address: School of Behavioural and Health Sciences
Australian Catholic University
1100 Nudgee Road, Banyo, QLD 4014, Australia

Email: Kassia.Beetham@acu.edu.au

Supplementary Table 1 Descriptive statistics for weekly exercise duration ($min \cdot wk^{-1}$) across phases

Phase	n	Mean	SD	Median	Q1 - Q3	Min	Max
Pre-conception	18	416	299	327	177 - 622	0	1,313
Pregnancy	21	351	266	292	153 - 465	20	1,437
Postpartum	19	278	295	188	153 - 465	0	1,301

n refers to the number of participants who provided exercise data for each phase

Supplementary Table 2 Descriptive statistics for average exercise session duration ($min \cdot session^{-1}$) across phases.

Phase	n	Mean	SD	Median	Q1 - Q3	Min	Max
Pre-conception	19	53	31	41	33 - 59	12	217
Pregnancy	21	50	33	41	31 - 56	11	248
Postpartum	20	46	21	42	31 - 56	10	134

n refers to the number of participants who provided exercise data for each phase

Supplementary Table 3 Descriptive statistics for weekly exercise frequency ($sessions \cdot wk^{-1}$) across phases.

Phase	n	Mean	SD	Median	Q1 - Q3	Min	Max
Pre-conception	18	8	6	7	4 - 11	0	34
Pregnancy	21	7	4	7	4 - 10	1	25
Postpartum	19	5	4	4	4 - 10	0	20

n refers to the number of participants who provided exercise data for each phase

Supplementary Table 4 Descriptive statistics for average weekly aerobic exercise intensity (% age-predicted HR_{max}) across phase.

Phase	n	Mean	SD	Median	Q1 - Q3	Min	Max
Pre-conception	17	62	13	59	52 - 74	41	96
Pregnancy	18	62	10	59	54 - 69	42	101
Postpartum	17	58	10	56	54 - 69	41	95

n refers to the number of participants who provided exercise heart rate data for each phase

Light-intensity, 40 < 55% HR_{max}; Moderate-intensity, 55 < 70% HR_{max}; Vigorous-intensity, 70 < 90% HR_{max}; High-intensity, ≥ 90% HR_{max}

Supplementary Table 5 Descriptive statistics for weekly aerobic exercise volume (MET-min·wk^{-1}) across phases.

Phase	n	Mean	SD	Median	Q1 - Q3	Min	Max
Pre-conception	17	1,481	1,472	1,039	504 - 1835	0	9,162
Pregnancy	18	938	924	685	264 - 1300	0	6,413
Postpartum	17	672	875	258	264 - 1300	0	3,785

n refers to the number of participants who provided exercise data for each phase
