# Supplementary material for: Patterns in exercise behaviour across pregnancy: a retrospective cohort study of physically active individuals from pre-conception to postpartum
Source: Eur J Appl Physiol. 2026 Feb 25;126(6):3407–27. doi: 10.1007/s00421-026-06160-6 (PMC13287166; doi:10.1007/s00421-026-06160-6)
Supplement: Supplementary file 2 — Supplementary Material 2 [file 421_2026_6160_MOESM2_ESM.pdf]

Online Resource 2: Devices used by participants to record heart rate during exercise

Article title: Patterns in exercise behaviour across pregnancy: a retrospective cohort study of physically active individuals from pre-conception to postpartum

Journal name: European Journal of Applied Physiology

Authors: Kate L Oxnard[1,2, 3], Rich D Johnston[1,2,4], Jemima G Spathis[1], Evelyn B Parr[5], Kassia S Beetham[1,2]

[1]School of Health and Behavioural Sciences, Australian Catholic University, 1100 Nudgee Road, Banyo, Brisbane, Queensland 4012, Australia

[2]Sports Performance, Recovery, Injury and New Technologies (SPRINT) Research Centre, Australian Catholic University, 1100 Nudgee Road, Banyo, Brisbane, Queensland, 4014, Australia

[3]College of Healthcare Sciences, James Cook University, 1 James Cook Drive, Douglas, Townsville, Queensland, 4814, Australia

[4]Carnegie Applied Rugby Research (CARR) Centre, Carnegie School of Sport, Leeds Beckett University, Leeds, United Kingdom

[5]Mary MacKillop Institute for Health Research, Australian Catholic University, Level 3, 250 Victoria Parade, Fitzroy, VIC 3065, Australia

Corresponding Author: Kassia Beetham

Address: School of Behavioural and Health Sciences

Australian Catholic University

1100 Nudgee Road, Banyo, QLD 4014, Australia

Email: Kassia.Beetham@acu.edu.au

Supplementary Table 6 Devices used by participants to record heart rate during exercise.

Device	*n* (%)
Apple Watch Series 2	1
Apple Watch Series 7	1
Apple Watch (no model specified)	2
Fitbit Luxe	1
Fitbit (no model specified)	1
Garmin Descent MK2	2
Garmin Fenix	2
Garmin Forerunner 45	1
Garmin Vivoactive 3	2
Garmin Vivoactive 4	1
Garmin (no model specified)	1
WHOOP (no model specified)	2
Withings Steel HR Sport	1
Not reported	3
