# Supplementary material for: Patterns in exercise behaviour across pregnancy: a retrospective cohort study of physically active individuals from pre-conception to postpartum
Source: Eur J Appl Physiol. 2026 Feb 25;126(6):3407–27. doi: 10.1007/s00421-026-06160-6 (PMC13287166; doi:10.1007/s00421-026-06160-6)
Supplement: Supplementary file 3 — Supplementary Material 3 [file 421_2026_6160_MOESM3_ESM.pdf]

Online Resource 3: Characteristics of participants who engaged in high-intensity antenatal exercise

Article title: Patterns in exercise behaviour across pregnancy: a retrospective cohort study of physically active individuals from pre-conception to postpartum

Journal name: European Journal of Applied Physiology

Authors: Kate L Oxnard[1,2, 3], Rich D Johnston[1,2,4], Jemima G Spathis[1], Evelyn B Parr[5], Kassia S Beetham[1,2]

[1]School of Health and Behavioural Sciences, Australian Catholic University, 1100 Nudgee Road, Banyo, Brisbane, Queensland 4012, Australia

[2]Sports Performance, Recovery, Injury and New Technologies (SPRINT) Research Centre, Australian Catholic University, 1100 Nudgee Road, Banyo, Brisbane, Queensland, 4014, Australia

[3]College of Healthcare Sciences, James Cook University, 1 James Cook Drive, Douglas, Townsville, Queensland, 4814, Australia

[4]Carnegie Applied Rugby Research (CARR) Centre, Carnegie School of Sport, Leeds Beckett University, Leeds, United Kingdom

[5]Mary MacKillop Institute for Health Research, Australian Catholic University, Level 3, 250 Victoria Parade, Fitzroy, VIC 3065, Australia

Corresponding Author: Kassia Beetham

Address: School of Behavioural and Health Sciences

Australian Catholic University

1100 Nudgee Road, Banyo, QLD 4014, Australia

Email: Kassia.Beetham@acu.edu.au

Supplementary Table 7 Characteristics of participants who engaged in high-intensity exercise during pregnancy

Characteristics	**Overall** (n=5)		
	**n** (%)	**Mean** (SD)	**Median** [Min, Max]
**Age at survey completion** (y)	-	36 (2.45)	36 [32, 38]
**First pregnancy**	1 (20)	-	-
**Mode of conception**	-	-	-
Spontaneous / unassisted	4 (80)	-	-
Assisted reproductive technology	1 (20)	-	-
**Region of residence**	-	-	-
Australia	2 (40)	-	-
Europe	1 (20)	-	-
North or Central America	2 (40)	-	-
**Marital status**	-	-	-
Married	5 (100)	-	-
**Highest level of education**	-	-	-
Bachelor's degree	2 (40)	-	-
Master's degree	2 (40)	-	-
Doctoral degree	1 (20)	-	-
**Pre-conception BMI**	-	24.02 (5.08)	21.59 [21.23, 31.64]
**Gestational weight gain** (kg)	-	9.11 (4.85)	7.50 [5.44, 16.00]
Insufficient	2 (40)	-	-
Within normal limits	1 (20)	-	-
'Excessive'	1 (20)	-	-
Not reported	1 (20)	-	-
**Competed in sport in the six months pre-conception**	2 (40)	-	-
Main competitive sport(s)	-	-	-
Running	2 (40)	-	-
Highest ever level of competition	-	-	-
Fun run or charity event	1 (20)	-	-
Local level	1 (20)	-	-

BMI, Body mass index.

Supplementary Table 8 Birth outcomes for participants who engaged in high-intensity exercise during pregnancy

Characteristics	**Overall** (n=5)		
	**n**<br>(%)	**Mean**<br>(SD)	**Median**<br>[Min, Max]
**Infant sex**	-	-	-
Female	3 (60)	-	-
Male	2 (40)	-	-
**Birth weight** (g)	-	2994 (326.74)	2980 [2551, 3400]
Within normal limits	5 (100)	-	-
**Gestational age at birth** (wk)	-	37.6 (1.67)	38 [35, 39)
Late preterm	1 (20)	-	-
Early term	2 (40)	-	-
Full term	2 (40)	-	-
**Mode of delivery**	-	-	-
Vaginal	5 (100)	-	-
**Delivery interventions**	-	-	-
Forceps	1 (20)	-	-
Episiotomy	2 (40)	-	-
Induction	2 (40)	-	-
Epidural	2 (40)	-	-
No intervention	1 (20)	-	-
**Birth complications**	-	-	-
Prolonged labour	1 (20)	-	-
Fetal distress	1 (20)	-	-
Perineal tear	1 (20)	-	-
None of the above	2 (40)	-	-

wk, Weeks
